# Evaluating the performance of GPT-3.5, GPT-4, and GPT-4o in the Chinese National Medical Licensing Examination

**DOI:** 10.1038/s41598-025-98949-2

**Published:** 2025-04-23

**Authors:** Dingyuan Luo, Mengke Liu, Runyuan Yu, Yulian Liu, Wenjun Jiang, Qi Fan, Naifeng Kuang, Qiang Gao, Tao Yin, Zuncheng Zheng

**Affiliations:** 1 Department of Rehabilitation Medicine Center, Affiliated Tai’an Central Hospital, Qingdao University, No. 29, Longtan Road, Taishan District, Tai’an City, 271000 Shandong China; 2 Department of Radiology, Affiliated Shandong Provincial Hospital, Shandong First Medical University, Jinan, 250021 Shandong China

**Keywords:** ChatGPT, Large Language models, Artificial intelligence, Medical licensing examination, Medical education, Health care, Medical research

## Abstract

This study aims to compare and evaluate the performance of GPT-3.5, GPT-4, and GPT-4o in the 2020 and 2021 Chinese National Medical Licensing Examination (NMLE), exploring their potential value in medical education and clinical applications. Six hundred original test questions from the 2020 and 2021 NMLE (covering five types of questions) were selected and input into GPT-3.5, GPT-4, and GPT-4o for response. The accuracy of the models across different question types and units was recorded and analyzed. Statistical methods were employed to compare the performance differences among the three models. GPT-4o demonstrated significantly higher overall accuracy than GPT-4 and GPT-3.5 (*P* < 0.001). In the 2020 and 2021 exams, GPT-4o achieved accuracy rates of 84.2% and 88.2%, respectively, with the highest accuracy observed in questions related to the digestive system (Unit 3), reaching 94.75%. GPT-4 showed moderate performance, while GPT − 3.5 had the lowest accuracy. Additionally, GPT-4o exhibited a clear advantage in complex question formats, such as case analysis questions (A3/A4 type) and standard matching questions (B1 type). GPT-4o outperformed its predecessors in the NMLE, demonstrating exceptional comprehension and problem-solving abilities in non-English medical examinations. This study provides important insights into the application and promotion of generative AI in medical education and clinical practice.

## Introduction

ChatGPT, a large language model (LLM) developed by OpenAI and released at the end of 2022, can understand and process natural language inputs. ChatGPT simulates human-like interactive conversations by leveraging extensive databases, deep learning, and machine learning^1,2^. Its emergence is regarded as a significant advancement in the field of cognitive intelligence, with increasing attention drawn to its potential applications in medicine^3,4^. In clinical practice, healthcare professionals can leverage LLMs such as ChatGPT to substantially enhance efficiency across multiple domains, including clinical diagnosis^5,6^, medical record documentation^7^, medical imaging analysis^8–10^, and disease prediction^10–12^. ChatGPT is a versatile learning tool in medical education, providing medical students with rapid and concise responses to queries, facilitating the analysis of common errors in examination questions, and reinforcing knowledge comprehension through interactive teaching modalities^13^. Compared to traditional pedagogical approaches, this technology transcends temporal and spatial constraints, markedly improving students’ learning efficiency. Furthermore, ChatGPT integrates educational resources across diverse medical specialties, offering educators a consolidated teaching platform and enabling medical students to engage in interdisciplinary learning experiences^14^. Additionally, it delivers concise summaries of critical information for clinical research, empowering students to more effectively assimilate evidence-based medical knowledge amidst demanding academic workloads^15^.

However, the outputs generated by ChatGPT are not always accurate and may occasionally provide misleading information, potentially impacting users’ judgment. Consequently, recent studies have attempted to assess the model’s accuracy and reasoning abilities by testing it with various medical exam question banks. Early versions of ChatGPT have successfully passed the United States Medical Licensing Examination (USMLE)^16,17^, the Neurology Board Exam^18^, and the Orthopedic Training Exam^19^, achieving performance levels comparable to human experts in certain areas. Nevertheless, research indicates that ChatGPT encounters limitations when handling non-English medical exams, demonstrating reduced accuracy and logical consistency. For example, ChatGPT has not yet passed the Chinese National Medical Licensing Examination (NMLE)^20–22^, the Chinese Pharmacist and Nursing Licensing Exams^22^, or the Korean Medical Licensing Examination^23^. These discrepancies may stem from semantic and cultural differences and variations in exam content and national regulations.

On May 13, 2024, OpenAI released the latest version, GPT-4o, which enhances real-time reasoning capabilities across audio, visual, and text modalities, significantly improving multimodal interaction. This version allows users to communicate via text and upload images and audio, enabling the model to process and output multiple data types simultaneously. Additionally, GPT-4o has shown substantial improvements in multilingual comprehension and processing. Therefore, evaluating the performance of GPT-4o in addressing non-English medical questions is of great significance for advancing the medical industry and education in non-English-speaking countries.

In China, the NMLE is a comprehensive assessment of medical students’ professional competencies and a prerequisite for obtaining a medical license. The written component of the NMLE consists of multiple-choice questions (MCQs) across five formats: A1 (single-best answer questions), A2 (case summary-based questions), A3 (multiple-case-based questions), A4 (case series-based questions), and B1 (matching questions). The exam comprises approximately 600 questions distributed across four units. The first unit primarily evaluates foundational medical knowledge, while the remaining three units focus on clinical subjects, including internal medicine, surgery, gynecology, and pediatrics. The NMLE assesses the professional knowledge and basic skills required for clinical practice, representing a core qualification and fundamental requirement for medical practitioners.

While previous studies have evaluated the capabilities of GPT-4o through subspecialty examinations in fields such as dentistry^24^, emergency medicine^25^, and rheumatology^26^, systematic investigations targeting the Chinese NMLE remain scarce. To address this gap, the present study aims to systematically compare the performance of GPT-3.5, GPT-4, and GPT-4o on the NMLE, elucidating the relative strengths and weaknesses of these model iterations in handling Chinese-language medical examinations. Furthermore, this study examines the performance of ChatGPT across different question types and examination sections, providing a comprehensive analysis of its capabilities. As the first systematic evaluation of GPT-4o in the context of the Chinese NMLE, this research not only fills a critical knowledge gap regarding the application of LLMs in Chinese-language licensing examinations but also offers valuable insights for optimizing LLMs in medical education and clinical practice in non-English-speaking countries.

## Methods

### Data collection

We selected the original test questions from the 2020 and 2021 Chinese NMLE, with each set consisting of four units containing 150 questions per unit, totaling 600. Each unit comprised various question types, including 228 A1-type questions (38.0%), 198 A2-type questions (33.0%), 58 A3/A4-type questions (9.7%), and 71 B-type questions (11.8%). Each question was weighted equally at one point. According to NMLE regulations, a total score of 360 or above is considered a passing grade.

GPT-3.5, GPT-4, and the latest GPT-4o models were employed to respond to the 2020 and 2021 NMLE test questions.

### Study design

The testing was conducted between June 10, 2024, and June 30, 2024. These models were accessed via the official OpenAI website’s chat interface rather than an Application Programming Interface. All questions were input in Chinese, with responses provided in Chinese. Each question was entered only once, and the responses were recorded in real-time. The temperature parameter was fixed at 0.7 to minimize variability in the responses. All 600 multiple-choice questions from the 2020 and 2021 NMLE were sequentially input into GPT-3.5, GPT-4, and GPT-4o, following the original order in the exam. Each model was instructed to respond as a medical professional, selecting the most appropriate answer from the provided options. Only one final answer was allowed for each question, regardless of whether it aligned with the expected standard.

To assess the compliance and accuracy of ChatGPT’s responses, an answer was marked as “correct” only if the model explicitly provided the correct option. If ChatGPT refused to answer or selected an incorrect option, the response was recorded as “incorrect.” In addition, the correct and incorrect responses were thoroughly analyzed to evaluate each model’s overall accuracy and performance characteristics. The responses of ChatGPT were recorded for each unit and question type, and accuracy was calculated by comparing the responses to the standard answers.

A comparative analysis of the test results across different models was conducted to explore the practicality and reliability of ChatGPT in applying medical knowledge. The potential applications of ChatGPT in medical education, disease diagnosis, and treatment were further analyzed based on the findings.

### Statistical analysis

Data were collected and organized using Microsoft Excel 16 (Microsoft, USA) to calculate accuracy and score rates as percentages. Visual representations of the results were generated using Excel to enhance the interpretability of the findings.

Statistical analysis was conducted using SPSS 23 (IBM, Armonk, NY, USA). A chi-square test was applied to compare the accuracy of responses among GPT-3.5, GPT-4, and GPT-4o. Pairwise comparisons between the three models were performed to analyze categorical data differences. Additionally, chi-square tests were employed to evaluate the accuracy of different units and question types to ensure rigor and reliability in the results.

## Results

### Overall analysis

In the 2020 NMLE test, the overall accuracy rates of GPT-3.5, GPT-4, and GPT-4o were 50.5%, 74.7%, and 84.2%, respectively. The accuracy rates for the 2021 NMLE test were 50.8%, 73.2%, and 88.2%. The differences in accuracy between the models for 2020 and 2021 were statistically significant (*P* < 0.001). GPT-4o demonstrated significantly higher accuracy than GPT-3.5 and GPT-4, while GPT-4 also significantly outperformed GPT-3.5 (*P* < 0.001). Detailed results are presented in Tables 1 and 2.

**Table 1:** Overall accuracy performance in 2020.

	2020						Pairwise comparisons					
	GPT-3.5	GPT-4	GPT-4o	Cramers V	c	P	3.5 vs. 4		4 vs. 4o		3.5 vs. 4o	
							c	P	c	P	c	P
Total questions	303/600(0.505)	448/600(0.746)	505/600(0.841)	0.309	171.442	< 0.001	74.822	< 0.001	16.563	< 0.001	154.592	< 0.001
Type of questions												
A1	117/228(0.513)	182/228(0.798)	194/228(0.851)	0.331	74.805	< 0.001	41.041	< 0.001	2.183	0.140	59.954	< 0.001
A2	104/198(0.525)	146/198(0.737)	159/198(0.803)	0.256	38.922	< 0.001	19.138	< 0.001	2.411	0.120	34.246	< 0.001
A3/A4	58/105(0.552)	72/105(0.686)	91/105(0.867)	0.281	24.959	< 0.001	3.958	0.047	9.896	0.002	25.161	< 0.001
B1	24/71(0.338)	48/71(0.676)	61/71(0.859)	0.446	42.320	< 0.001	16.229	< 0.001	6.672	0.010	40.123	< 0.001
Unit of questions												
Unit 1	81/150(0.540)	113/150(0.753)	117/150(0.780)	0.232	24.317	< 0.001	14.939	< 0.001	0.298	0.585	19.251	< 0.001
Unit 2	78/150(0.520)	108/150(0.720)	134/150(0.893)	0.337	50.971	< 0.001	12.733	< 0.001	14.449	< 0.001	50.429	< 0.001
Unit 3	77/150(0.513)	112/150(0.747)	123/150(0.820)	0.284	36.183	< 0.001	17.518	< 0.001	2.376	0.123	31.740	< 0.001
Unit 4	67/150(0.447)	115/150(0.767)	131/150(0.873)	0.394	69.849	< 0.001	32.185	< 0.001	5.781	0.016	60.844	< 0.001

**Table 2:** Overall accuracy performance in 2021.

	2021						Pairwise comparisons					
	GPT-3.5	GPT-4	GPT-4o	Cramers V	c	P	3.5 vs. 4		4 vs. 4o		3.5 vs. 4o	
							c	P	c	P	c	P
Total questions	305/600(0.508)	434/600(0.723)	529/600(0.882)	0.335	202.372	< 0.001	58.616	< 0.001	47.452	< 0.001	197.256	< 0.001
Type of questions												
A1	117/225(0.520)	165/225(0.733)	202/225(0.898)	0.343	79.574	< 0.001	21.884	< 0.001	20.224	< 0.001	77.802	< 0.001
A2	104/206(0.505)	154/206(0.748)	181/206(0.878)	0.341	72.023	< 0.001	25.924	< 0.001	11.644	0.001	67.489	< 0.001
A3/A4	52/89(0.584)	68/89(0.764)	78/89(0.876)	0.275	20.169	< 0.001	6.547	0.011	3.810	0.051	19.283	< 0.001
B1	32/80(0.400)	47/80(0.588)	68/80(0.850)	0.379	34.444	< 0.001	5.626	0.018	13.635	< 0.001	34.560	< 0.001
Unit of questions												
Unit 1	80/150(0.533)	114/150(0.760)	131/150(0.873)	0.316	44.817	< 0.001	16.864	< 0.001	6.434	0.001	41.552	< 0.001
Unit 2	80/150(0.533)	107/150(0.713)	125/150(0.833)	0.276	32.170	< 0.001	10.350	0.001	6.161	0.013	31.194	< 0.001
Unit 3	69/150(0.460)	108/150(0.720)	142/150(0.947)	0.438	86.212	< 0.001	20.959	< 0.001	27.744	< 0.001	85.132	< 0.001
Unit 4	76/150(0.507)	105/150(0.70)	131/150(0.873)	0.325	47.471	< 0.001	11.714	0.001	13.427	< 0.001	47.140	< 0.001

### Analysis by question type

**Fig. 1:**
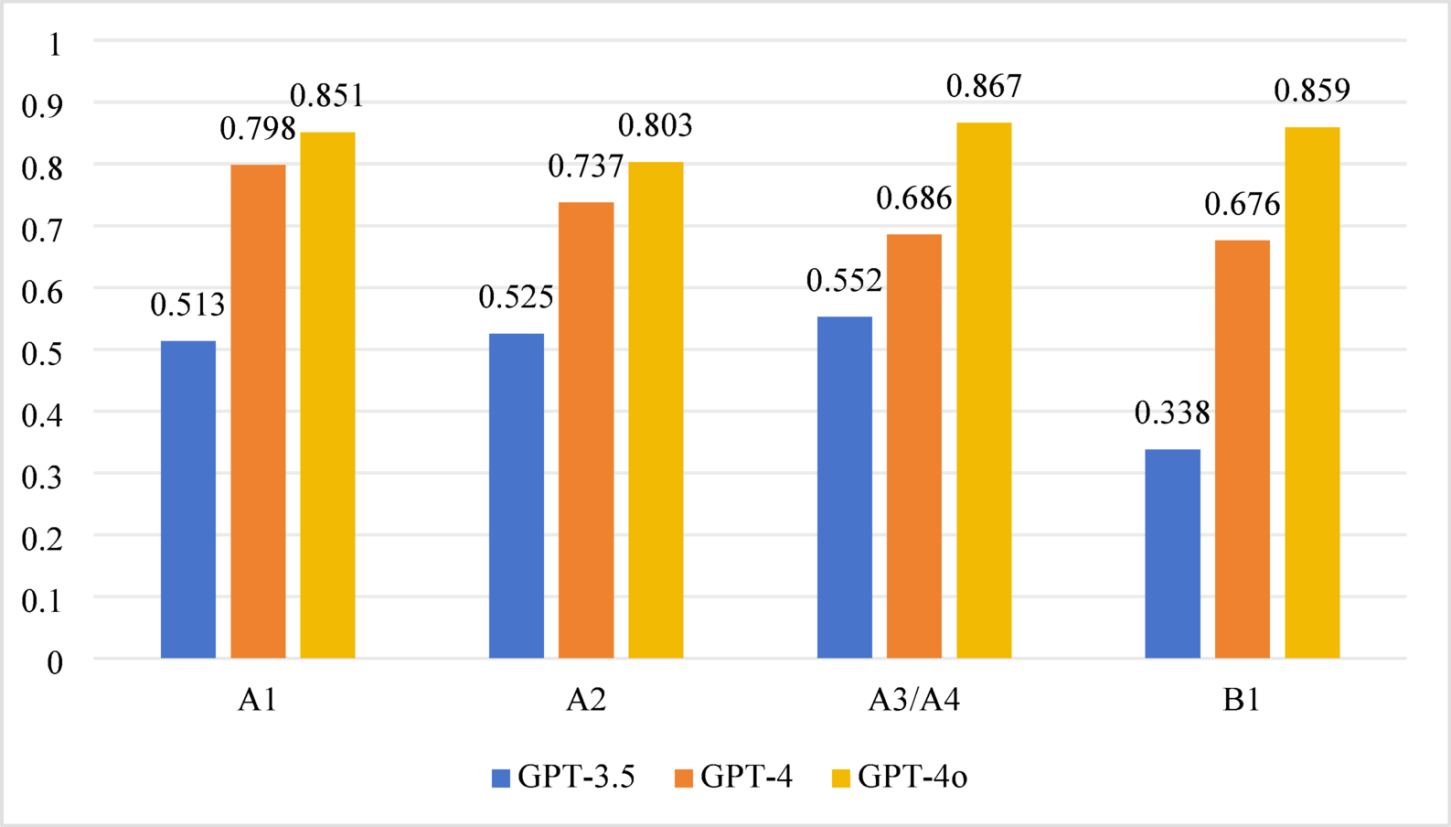
Accuracy performance of different question types in 2020.

For the 2020 test, significant differences in accuracy were observed among GPT-3.5, GPT-4, and GPT-4o across all question types (A1, A2, A3/A4, B1) (*P* < 0.001). The pairwise comparison results were as follows:

- *GPT-3.5 vs. GPT-4* Significant differences were found in the accuracy rates for the A1, A2, and B1 question types (*P* < 0.0167), while no significant difference was observed for A3/A4 questions (*P* > 0.0167).
- *GPT-4 vs. GPT-4o* Significant differences were noted in A3/A4 and B1 question types (*P* < 0.0167), but no significant difference was detected in A1 and A2 question types (*P* > 0.0167).
- *GPT-3.5 vs. GPT-4o* Significant differences were observed across all question types (*P* < 0.0167). Detailed results are shown in Table 1; Fig. 1.

**Fig. 2:**
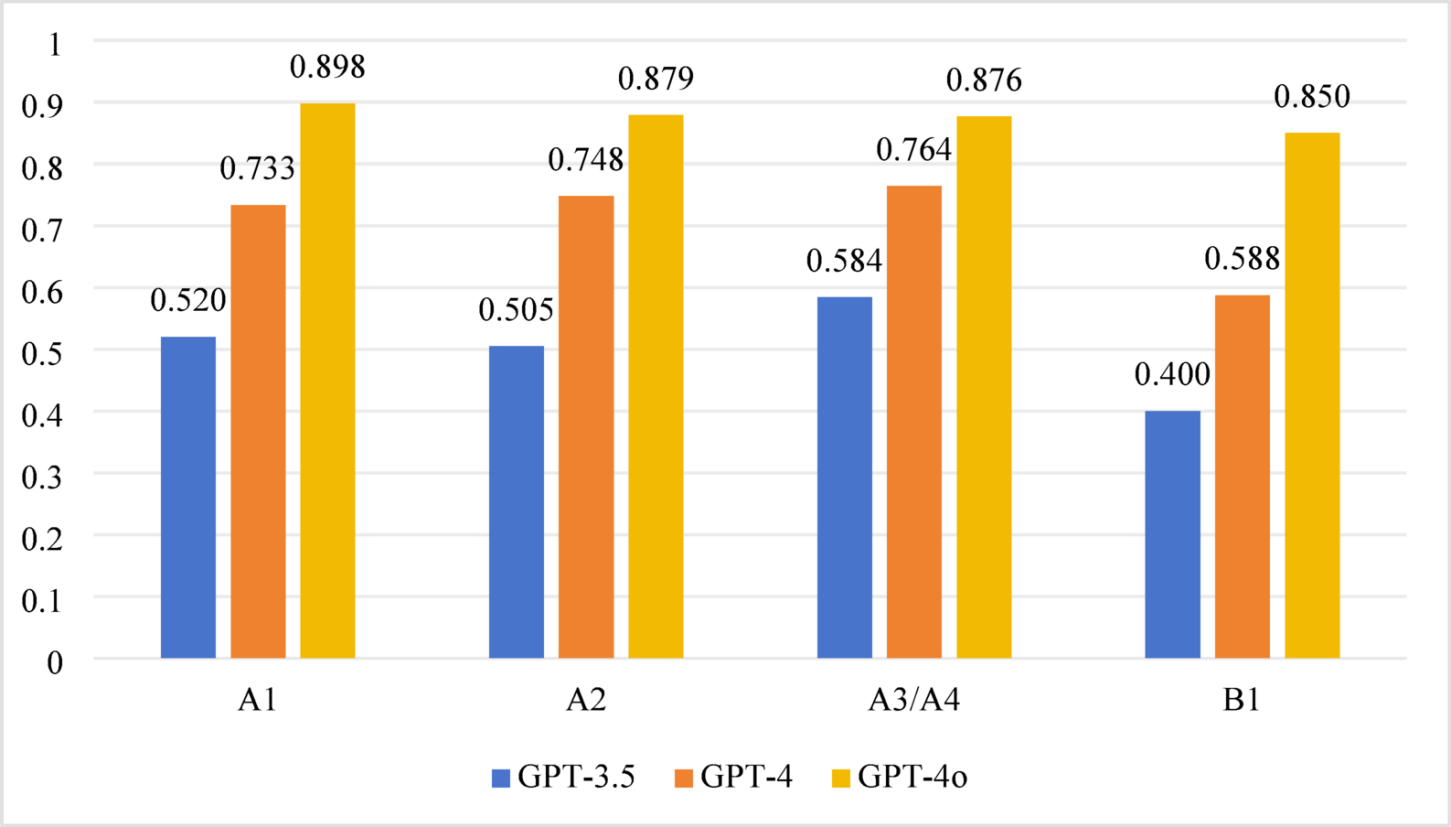
Accuracy performance of different question types in 2021.

In the 2021 test, significant differences in accuracy were also found across all question types among the three models (*P* < 0.001). Pairwise comparisons revealed:

- *GPT-3.5 vs. GPT-4* Significant differences were observed for A1, A2, and A3/A4 question types (*P* < 0.0167), with no significant difference for B1 questions (*P* > 0.0167).
- *GPT-4 vs. GPT-4o and GPT-3.5 vs. GPT-4o* Significant differences were observed across all question types (*P* < 0.0167). Detailed results are shown in Table 2; Fig. 2.

### Analysis by unit

**Fig. 3:**
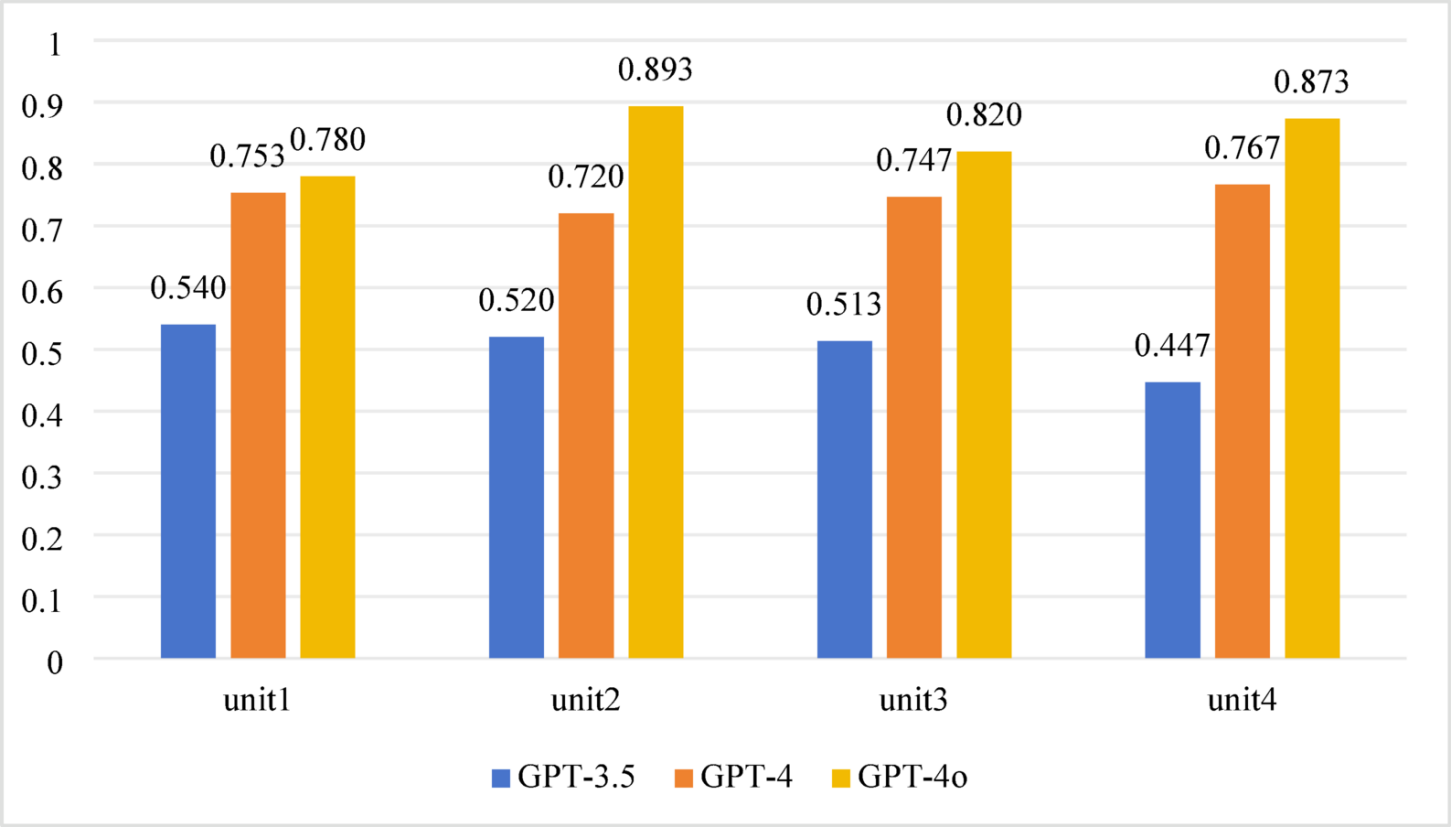
Accuracy performance of 4 units in 2020.

For the 2020 test, significant differences in accuracy among GPT-3.5, GPT-4, and GPT-4o were found across all units (Unit 1, Unit 2, Unit 3, Unit 4) (*P* < 0.001). The pairwise comparison results were as follows:

- *GPT-3.5 vs. GPT-4 and GPT-3.5 vs. GPT-4o* Significant differences were observed across all units (*P* < 0.001).
- *GPT-4 vs. GPT-4o* Significant differences were noted in Unit 2 and Unit 4 (*P* < 0.0167), while no significant differences were detected in Unit 1 and Unit 3 (*P* > 0.0167). Detailed results are provided in Table 1; Fig. 3.

**Fig. 4:**
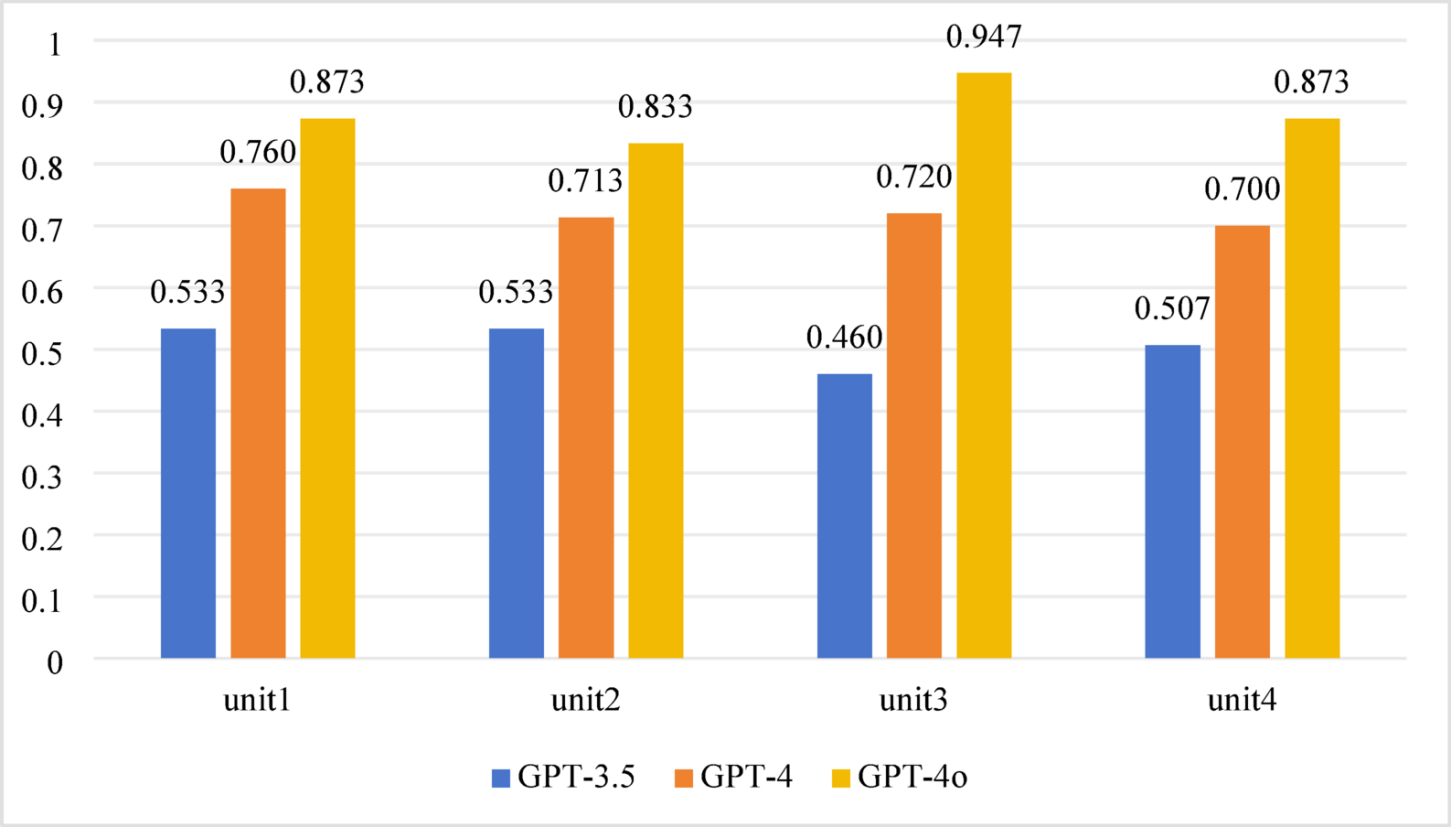
Accuracy performance of 4 units in 2021.

For the 2021 test, significant differences in accuracy were observed across all units among the three models (*P* < 0.001). Pairwise comparisons showed significant differences in accuracy across all units for each model pair (*P* < 0.0167). Detailed results are presented in Table 2; Fig. 4.

## Discussion

This study compares the performance of GPT-3.5, GPT-4, and GPT-4o on the 2020 and 2021 Chinese NMLE, focusing on the enhanced accuracy and reliability of GPT-4o in answering medical questions. The results indicate that GPT-4o achieved significantly higher accuracy in the 2020 and 2021 NMLE than GPT-3.5 and GPT-4. In subgroup analyses by question type and unit, GPT-4o consistently demonstrated superior performance. While GPT-4 showed competitive results in specific modules, it did not surpass GPT-4o, and both models significantly outperformed GPT-3.5.

China, a developing country with over 1.4 billion people, reported approximately 9.56 billion medical visits in 2023, with total healthcare expenditures reaching 8.4 trillion RMB. Despite having around 4.78 million licensed and assistant physicians, physician distribution and availability disparities persist. The NMLE, organized and standardized by the National Medical Examination Center, is a pivotal national licensing exam for medical practitioners in China, serving as a critical gateway to medical licensure. Therefore, the NMLE was selected as the benchmark for evaluating the application of ChatGPT in medical education, providing insights into its future role in healthcare development.

ChatGPT models, developed through deep learning techniques and extensive datasets, excel in efficient information retrieval and language organization. According to OpenAI, GPT-3.5, released in November 2022, contains approximately 175 billion parameters and is trained on data up to September 2021. GPT-4, released in 2023, expanded to one trillion parameters, while GPT-4o, launched on May 14, 2024, has 1.2 trillion parameters and is trained on data up to December 2023. To minimize the impact of out-of-database information on GPT-3.5’s performance, we selected the 2020 and 2021 NMLE tests for evaluation.

In the 2020 and 2021 exams, GPT-4o demonstrated the highest overall accuracy, followed by GPT-4, with GPT-3.5 performing the lowest. GPT-4 and GPT-4o achieved accuracy rates exceeding 60%, surpassing the NMLE passing threshold. According to official NMLE data, the national pass rate typically ranges between 18% and 22%. Hence, the performance of GPT-4 and GPT-4o exceeds that of most candidates, showcasing their remarkable potential in medical problem-solving and analysis.

While GPT-3.5 has passed the United States Medical Licensing Examination (USMLE)^16,17^, it has not succeeded in the NMLE^20–22^. Wang et al. reported GPT-3.5 accuracy rates of 47% and 45.8% for the 2020 and 2021 NMLE, attributing the discrepancy to differences in medical policies and epidemiological data between China and the United States^20^. Our findings corroborate these results, with GPT-3.5 achieving accuracy rates of 50.5% (2020) and 50.8% (2021), reflecting slight improvements potentially linked to operational details or the model’s inherent randomness. This also suggests that deep learning and model training enhance performance over time.

Zong et al. tested GPT-3.5 on the 2017–2021 NMLE, pharmacist, and nursing exams in China, revealing that GPT-3.5 failed to meet the passing standard for all tests. The authors attributed this to ChatGPT’s English-centric training, highlighting challenges in understanding medical policies outside English-speaking regions. However, GPT-3.5 consistently scored above 50%, underscoring AI’s potential in medical education^22^. Fang et al. attempted to improve results by translating NMLE questions into English before inputting them into ChatGPT, but the outcomes showed no significant enhancement^27^. Another study found a 5% improvement in accuracy when the NMLE was translated into professional English^28^. This discrepancy may stem from translation quality, but semantic and cultural differences remain key challenges for AI in non-English medical exams.

The advent of GPT-4 has significantly improved the model’s ability to comprehend non-English languages. Takagi et al. found that GPT-4 outperformed GPT-3.5 in the Japanese Medical Licensing Examination, successfully passing the test^29^. Similarly, GPT-4 met the passing threshold for the Chinese Master’s Degree Entrance Examination in Clinical Medicine, achieving accuracy rates of 73.67%^27^ and 81.25%^28^ in the NMLE, aligning with our findings. A separate meta-analysis examined the performance of LLMs across dental licensing examinations in diverse linguistic and geographical contexts, revealing that GPT-4 holds potential in dental education and diagnosis, albeit with accuracy levels still falling below the threshold required for clinical applications^30^. Our study demonstrates that GPT-4o significantly improved performance, achieving overall accuracy rates of 84.1% (2020) and 88.2% (2021). Ebel et al. found that GPT-4o passed the European Board of Interventional Radiology (EBIR) mock written exam, a qualification often associated with expert-level knowledge in interventional radiology^31^. Moreover, GPT-4o can generate exam questions at varying difficulty levels, offering valuable training and assessment tools for radiology residents and medical students^31^.

These findings suggest that LLMs could, in the future, be integrated into medical education within academic institutions and professional training for clinicians. Nevertheless, the responses generated by AI are not invariably accurate. First, GPT-4o may provide seemingly rational analyses even for incorrect answers, potentially misleading users. Second, we observed instances where GPT-4o selected the correct answer but provided flawed reasoning. For example, in an A2-type cardiology question, GPT-4o accurately identified the optimal therapeutic agent but exhibited errors in classifying the type of arrhythmia. Such discrepancies may stem from limitations in the underlying database and variations in medical theories, cultural contexts, and legal regulations across different countries. Consequently, when employing LLMs to address medical questions, it is imperative to critically evaluate the validity of their responses and avoid over-reliance on their outputs.

### Comparative analysis of question types

GPT-4o consistently outperformed GPT-4 across different question types, while GPT-4 generally exceeded GPT-3.5 in most cases. However, in case analysis questions (A3/A4) for the 2020 exam and standard matching questions (B1) for the 2021 exam, the accuracy difference between GPT-4 and GPT − 3.5 was not statistically significant.

A3 and A4 questions, collectively called case analysis questions, assess the ability to analyze clinical scenarios comprehensively. A3-type questions involve analyzing scenarios based on a single patient, with 2–3 related questions requiring independent judgment. A4-type questions are more complex, providing multi-level information as patient conditions unfold, necessitating deeper case analysis. These questions demand contextual understanding and diagnostic reasoning, posing significant challenges to ChatGPT’s ability to interpret and process information. B1-type questions feature an innovative format, where five options are used for at least two questions, testing the candidate’s ability to make the best selection. Wang et al. highlighted GPT-3.5’s subpar performance in case analysis questions^20^, while Li et al. found that multiple-choice questions appeared to be a weak point for both GPT-3.5 and GPT-4, with the lowest scores compared to other question types^32^. Takagi et al. observed that GPT-3.5’s accuracy for difficult questions was only 33.3%, whereas GPT-4’s increased by 40%, surpassing examinees’ accuracy by 17%^29^.

Our study corroborates these findings, indicating that GPT-4 and GPT-3.5 struggle with complex question types. GPT-3.5 performed the worst on B1-type questions, with 33.8% and 40% accuracy rates in 2020 and 2021, respectively. GPT-4 achieved a 58.8% accuracy rate for B1-type questions in 2021, the only instance where its accuracy fell below 60% across all question types and units. Additionally, GPT-4 showed poor performance in 2020 A3/A4-type questions, with no significant difference compared to GPT − 3.5.

These findings highlight the challenges B1-type and A3/A4-type questions pose for ChatGPT’s processing and analytical capabilities. However, GPT-4o demonstrated superior performance across all question types, achieving over 80% accuracy, with case analysis and standard matching questions exceeding 85%. However, as the written component of the NMLE does not include image recognition questions, the ability of GPT-4o to interpret Chinese-language electrocardiograms and imaging-related questions remains to be further validated.

### Comparative analysis by unit

GPT-4o consistently outperformed GPT-4 across units, particularly in 2021, where all units except Unit 1 and Unit 3 exhibited higher accuracy. Both GPT-4o and GPT-4 showed significantly higher accuracy than GPT-3.5.

- **Unit 1** primarily covers foundational subjects, focusing on A1-type questions emphasizing memorization.
- **Unit 2** assesses cardiovascular, urological, and musculoskeletal systems, with diagnostic, auxiliary examination, and treatment-related content contributing over 20 points each.
- **Unit 3** focuses on the digestive and respiratory systems.
- **Unit 4** involves the female reproductive, pediatric, and neurological/psychiatric systems.

We found that GPT-4o achieved its highest accuracy rate of 94.7% in Unit 3 of the 2021 examination, excelling in digestive and respiratory systems questions. However, its performance in Unit 3 of the 2020 examination was relatively modest (82%). In Unit 2 of the 2020 examination (covering cardiovascular, urinary, and musculoskeletal systems), GPT-4o achieved the second-highest accuracy rate of 89.3%, while it performed least effectively in Unit 1 (basic sciences), with an accuracy rate of 78%. The performance of GPT-4 in basic sciences and the digestive and respiratory systems was comparable to that of GPT-4o (*P* ≥ 0.001). In Unit 4 of the 2020 examination (covering female reproduction, pediatrics, and neuropsychiatry), GPT-4 achieved its highest accuracy rate of 76.7%, which was lower than the lowest accuracy rate of GPT-4o (78%), highlighting a performance disparity between the two models. The variation in accuracy rates of LLMs across different units reflects their differing capabilities across specialties, though it may also be influenced by variations in question difficulty across years. Additionally, the relatively small number of questions in each subspecialty may limit the ability to capture LLMs’ true proficiency in these domains fully. Lin et al. compared GPT-4o, Claude-3.5 Sonnet, and Gemini Advanced on Taiwan’s internal medicine exam, finding Claude-3.5 Sonnet excelled in psychiatry and nephrology. At the same time, GPT-4o achieved 97.1% accuracy in hematology and oncology, exceptionally outperforming image-based questions. Conversely, Gemini Advanced had the lowest overall accuracy but performed reasonably well in psychiatry (86.96%) and hematology/oncology (82.91%)^33^. Liu et al. categorized questions from the Japanese national medical examination into 21 specialties and compared the accuracy rates of LLMs in each specialty against their overall accuracy. They found that LLMs performed significantly worse in gastroenterology, hepatology, pulmonology, and hematology than their overall performance. Their analysis inferred that this disparity might be associated with the volume of academic publications in each specialty^34^. This methodological approach offers novel insights for future related studies. The specific capabilities of LLMs across various clinical specialties warrant further investigation by relevant professionals.

In studies focusing on image-based questions, Liu et al. observed that GPT-4o outperformed GPT-4, Gemini 1.5 Pro, and Claude 3 Opus in both image-based and non-image-based questions. However, image-based questions posed a greater challenge to LLMs, resulting in accuracy rates substantially lower than those for non-image-based questions^34^. Another study applied GPT-4, Gemini, GPT-4 Turbo, and GPT-4o to core cardiology exams, with GPT-4o delivering the best performance on text-based and image-based questions^35^. Fabijan et al. tested ChatGPT’s ability to evaluate scoliosis X-rays, finding that while identifying all scoliosis cases, its accuracy in determining curvature direction, type, and vertebral rotation was limited^36^. Nakao et al. discovered that adding image information to original Japanese medical licensing exam questions resulted in decreased accuracy for GPT-4 V, indicating that GPT-4 V struggles with medical image interpretation^37^.

These studies reveal that LLMs generally underperform in image-based questions compared to text-based questions^33–37^. Future research should enhance AI’s ability to analyze and interpret medical images, mainly refining AI’s computational capacity for image-specific details.

### Practical implications and limitations

In this study, we systematically evaluated the potential of ChatGPT, particularly its latest iteration, GPT-4o, in medical education and clinical practice. Our findings indicate notable feasibility in specific contexts but highlight significant limitations that necessitate careful consideration to ensure safe and effective application in the medical domain.

#### Advantages

First, in medical education, LLMs demonstrate considerable potential, particularly in facilitating knowledge acquisition and enhancing learning efficiency. ChatGPT can rapidly synthesize medical knowledge, saving medical students time otherwise spent consulting textbooks and literature, with its efficiency advantage being especially pronounced in foundational disciplines and broad knowledge domains. Studies have shown that LLMs can achieve high accuracy rates in certain tests; for instance, in a UK-based study, GPT-4 achieved 100% accuracy across a 20-question test^38^. A meta-analysis encompassing 45 studies on the performance of different ChatGPT versions in medical licensing examinations reported an overall accuracy rate of 81% for GPT-4^39^. In our study, GPT-4o exhibited high accuracy rates (nearly all above 85%) across complex question types, such as case analysis, standard matching questions, and simpler A1-type questions. This suggests that LLMs can be auxiliary tools to help students quickly grasp key concepts or address knowledge gaps, particularly in foundational knowledge and clinical simulation scenarios. In clinical practice, LLMs offer efficiency advantages in screening common diseases and managing diagnostics, rapidly analyzing complex data to provide preliminary diagnostic suggestions. In our study, GPT-4o achieved an accuracy rate of 94.7% in Unit 3 of the 2021 examination, with accuracy rates in other units all exceeding 83%, reflecting its robust performance across multiple specialties. This capability can help alleviate the workload of clinicians and enhance diagnostic efficiency, particularly in resource-limited settings.

#### Limitations

LLMs are a double-edged sword, and their potential risks and challenges warrant vigilance. First, ChatGPT is a machine learning system that autonomously learns from internet data and generates outputs after training on vast text datasets^14^. However, medical knowledge available online is not always reliable^40^. These unreliable data sources may compromise output performance and accuracy, leading to potential misinformation^41^. In our study, GPT-4o exhibited inaccuracies and deficiencies in certain highly specialized domains yet delivered responses in an “authoritative” tone, which could foster overconfidence among users. Medicine, a rigorous discipline tied to human lives, is particularly vulnerable to the consequences of misleading information, which could directly impair students’ learning outcomes, interfere with clinicians’ judgment, and pose potential risks to patient health. Second, researchers have observed a degree of randomness in GPT-4’s responses^39^, a phenomenon also noted in our study, where GPT-4o occasionally provided inconsistent answers to identical questions, significantly impacting user judgment. Third, over-reliance on LLMs may lead to an “answer dependency” phenomenon, stifling students’ independent thinking and critical reasoning skills. Students may become inclined to adopt ChatGPT’s suggestions directly, neglecting the need to master foundational knowledge and cultivate a spirit of inquiry. Finally, privacy protection and data security in clinical applications of LLMs are critical considerations. ChatGPT systems must rigorously safeguard patient information to prevent sensitive data breaches. Additionally, ChatGPT algorithms’ transparency and ethical implications require scrutiny to mitigate potential biases or misleading suggestions that could adversely affect patients. These considerations should form the foundation of cautious implementation in the medical application of ChatGPT, ensuring tangible and positive impacts on patients and healthcare professionals.

In summary, while LLMs demonstrate potential in medical education and clinical diagnostics, such as improving learning efficiency and aiding in the diagnosis of common diseases, their limitations—including knowledge inaccuracies, output randomness, risk of dependency, and ethical and privacy concerns—preclude their use as primary knowledge sources in medical education or standalone tools in clinical diagnostics. To maximize their benefits while mitigating risks, we recommend designing hybrid learning models in medical education that integrate LLMs, encouraging students to engage in critical reflection under AI assistance. In clinical practice, establishing secondary validation mechanisms for AI-driven decisions is essential, with healthcare professionals conducting necessary reviews to ensure clinical decisions genuinely benefit patient safety. Future research should further explore ways to enhance the accuracy and reliability of LLMs in highly specialized domains and localized contexts while refining associated ethical and regulatory frameworks to safeguard privacy and data security.

### Study limitations

Our study has several limitations.

#### Scope of analysis

This study primarily focuses on descriptive statistical comparisons of GPT-3.5, GPT-4, and GPT-4o in the NMLE. While predictive modeling could provide further insights into factors influencing AI accuracies, such as question complexity and domain-specific difficulty, such analyses were beyond the scope of this study. Future research should consider implementing logistic regression or other predictive models to explore the relationships between question characteristics and AI performance.

#### Lack of image-based questions

The study exclusively tested text-based questions, omitting image-based evaluations. Future studies should incorporate more diverse question types, including imaging case analysis, to assess ChatGPT’s multimodal performance.

#### AI advancement

The rapid advancement of AI technology introduces additional complexity and limitations to this study. Our research relies on data and technological capabilities available before 2024. With the swift progress of LLMs in natural language processing and specialized applications, future iterations will likely surpass current models’ performance. For instance, the newly released Deepseek in 2025 was not included in this study due to differences in its training environment, which is based on a Chinese-language database. In conclusion, the results of this study may not fully reflect the true performance of future AI models in medical examinations.

Despite these limitations, this study provides valuable insights into the evolving role of AI in medical education and professional licensing exams. Future research should expand question sets, enhance cross-language accuracy, and continuously refine assessment methodologies to reflect AI’s growing role in medical education and clinical applications.

## Conclusion

This study systematically evaluated the performance of GPT-3.5, GPT-4, and GPT-4o on the 2020 and 2021 Chinese NMLE. The results demonstrate that GPT-4o outperformed GPT-4 and GPT-3.5 in overall accuracy, complex question-solving, and multi-unit assessments. This finding highlights the potential of the latest generative AI model in addressing non-English medical problems.

Future research should incorporate a wider range of question types, broader coverage of medical disciplines, and multimodal data tests to comprehensively assess the potential of ChatGPT and other generative AI models in medical education and clinical practice.

GPT-4o strongly supports non-English medical education and professional licensing examinations, showcasing its promising role as an auxiliary tool in clinical diagnostics. However, its reliability and safety require further validation to establish a foundation for widespread application.

## Data Availability

All data generated or analyzed during this study are available from the corresponding author upon reasonable request.
